# Supplementary material for: Comparative analysis of adolescent pregnancy and delivery outcomes versus early adulthood pregnancy in the Asante Akim North District, Ghana
Source: PLOS Glob Public Health. 2025 Feb 24;5(2):e0004290. doi: 10.1371/journal.pgph.0004290 (PMC11849814; doi:10.1371/journal.pgph.0004290)
Supplement: S2 Table — (DOCX) [file pgph.0004290.s002.docx]

**Table 2b Other Young People Maternal Outcomes and/or Complications**

| **Indicator** | **Year** | | | **Total** |
| --- | --- | --- | --- | --- |
|  | **2018** | **2019** | **2020** |  |
| Ante partum haemorrhage | 0 | 2 | 0 | 3 |
| Breech presentation | 0 | 1 | 2 | 6 |
| Delayed first stage of labour | 1 | 8 | 1 | 16 |
| Delayed second stage of labour | 1 | 8 | 0 | 9 |
| Post datism | 0 | 10 | 4 | 17 |
