# Supplementary material for: Comparative analysis of adolescent pregnancy and delivery outcomes versus early adulthood pregnancy in the Asante Akim North District, Ghana
Source: PLOS Glob Public Health. 2025 Feb 24;5(2):e0004290. doi: 10.1371/journal.pgph.0004290 (PMC11849814; doi:10.1371/journal.pgph.0004290)
Supplement: S3 Table — (DOCX) [file pgph.0004290.s003.docx]

**Table 2c Association between Maternal Outcomes and Complications, and Maternal Age**

| **Indicators** | **Year & Maternal age** | | | **T** | **Year & Maternal age** | | | **T** | **Year & Maternal age** | | | **T** | **GT** | **Chi-square**  **Value** | **P-value** |
| --- | --- | --- | --- | --- | --- | --- | --- | --- | --- | --- | --- | --- | --- | --- | --- |
|  | **2018** | | |  | **2019** | | |  |  | **2020** |  |  |  |  |  |
|  | **10-14** | **15-19** | **20-24** |  | **10-14** | **15-19** | **20-24** |  | **10-14** | **15-19** | **20-24** |  |  |  |  |
| **Outcomes** | | | | | | | | | | | | | | | |
| `PPH | 2 | 5 | 11 | **18** | 0 | 5 | 4 | **9** | 0 | 6 | 4 | **10** | **37** | 11.573^a^ | <**0.001*** |
| Caesarean delivery | 0 | 12 | 18 | **30** | 0 | 9 | 14 | **23** | 0 | 10 | 9 | **19** | **72** | 0.481^a^ | **0.483** |
| Admission to hospital for a longer time (more than 2 days) | 1 | 19 | 14 | **34** | 0 | 11 | 16 | **27** | 0 | 10 | 9 | **19** | **80** | 0.264^a^ | **0.033*** |
| Surgical site infection | 0 | 4 | 2 | **6** | 0 | 2 | 4 | **6** | 0 | 3 | 1 | **4** | **16** | 0.137^a^ | **0.277** |
| Genital tract sepsis | 2 | 4 | 2 | **8** | 1 | 12 | 7 | **20** | 0 | 5 | 9 | **14** | **42** | 20.314^a^ | <**0.001*** |
| Uti/pneumonia, breast infection, infected episiotomy | 2 | 7 | 7 | **16** | 3 | 23 | 14 | **40** | 0 | 13 | 10 | **23** | **79** | 27.296^a^ | <**0.001*** |
| Maternal death | 1 | 0 | 1 | **2** | 0 | 0 | 0 | **0** | 0 | 1 | 0 | **1** | **3** | 32.211^a^ | <**0.001*** |
| **Complications** | | | | | | | | | | | | | | | |
| Hypertensive disorders of pregnancy | 0 | 2 | 6 | **8** | 0 | 4 | 14 | **18** | 0 | 4 | 5 | **9** | **35** | 0.152^a^ | **0.505** |
| Anaemia in pregnnacy | 5 | 60 | 32 | **97** | 1 | 72 | 63 | **136** | 0 | 51 | 39 | **90** | **323** | 4.410^a^ | <**0.001*** |
| Gestational DM | 0 | 0 | 1 | **1** | 0 | 1 | 8 | **9** | 0 | 0 | 0 | **0** | **10** | 0.015^a^ | **0.194** |
| Premature rupture of the membranes | 0 | 10 | 4 | **14** | 0 | 5 | 8 | **13** | 0 | 0 | 2 | **2** | **29** | 0.229^a^ | **0.250** |
| Placenta previa | 0 | 0 | 0 | **0** | 0 | 2 | 0 | **2** | 0 | 0 | 0 | **0** | **2** | 0.030^a^ | **0.184** |
| Other (specify) | 2 | 8 | 8 | **18** | 0 | 16 | 15 | **31** | 0 | 9 | 1 | **10** | **59** | 14.900^a^ | <**0.001*** |

**T = total for the year, GT = grand total for the 3 years (2018-2020), p-value set at <0.05**
