# Supplementary material for: Comparative analysis of adolescent pregnancy and delivery outcomes versus early adulthood pregnancy in the Asante Akim North District, Ghana
Source: PLOS Glob Public Health. 2025 Feb 24;5(2):e0004290. doi: 10.1371/journal.pgph.0004290 (PMC11849814; doi:10.1371/journal.pgph.0004290)
Supplement: Attachment — Submitted filename: Response to Reviewers.pdf [file pgph.0004290.s007.pdf]

c/o Amantenaman Health Centre

P.O. Box GG133

Agogo-Asante Akim North

Ghana

26th September 2024

Editor-in-Chief

PLOS Global Public Health

Dear Editor-in-Chief,

I am writing to submit my revised manuscript titled "**Comparative analysis of adolescent pregnancy and delivery outcomes versus early adulthood pregnancy in the Asante Akim North District, Ghana"** for consideration for publication in PLOS Global Public Health.

Response to Reviewers Comments on Manuscript Titled: Comparative analysis of adolescent pregnancy and delivery outcomes versus early adulthood pregnancy in the Asante Akim north district, Ghana

Abstract

Reviewers comments 1

Keywords: The keywords are appropriate and capture the main aspects of the study. However, they could be expanded to include terms like "maternal health," "reproductive health," and" delivery outcomes.

Response to Reviewer comment 1

Keywords: pregnancy outcomes, complications, teenagers, young adults, Asante Akim North District, maternal health, reproductive health, delivery outcomes, postpartum haemorrhage

Reviewer comment 2

Background: The background is generally clear but could benefit from more detail on the global and regional context of adolescent pregnancy, specifically in LMICs like Ghana. The sentence "Despite the evidence of maternal of adolescent pregnancies globally, little is known, especially in low-income countries" is somewhat awkward and could be rephrased for clarity.

Response to Reviewer comment 2

Globally, it is estimated that about 18 million girls under 20 years give birth every year. Sixteen million of these girls are aged 15–19 years, 2 million are below 15 years, and contribute nearly 11% of births worldwide (Nagandla & Kumar, 2020). High rate of adolescent pregnancies in Low and Middle-Income Countries (LMICs) has become a major threat to the health and safety of young female adolescents.

Reviewers comment 3

Objective: The objective is clearly stated and aligns with the research question. However, it could be more specific about the types of outcomes being compared (e.g., maternal and neonatal outcomes).

Response to Reviewer comment 3

This study assessed adolescent pregnancy and delivery of maternal and neonatal outcomes compared to young adults in the Asante Akim North District, Ghana.

Reviewers comment 4

Methods: The methodology is well-outlined, mentioning the study design, data collection period, and tools used for analysis. However, the sentence "Health facilities and participants were selected using a multi-stage sampling technique" could be expanded to explain the sampling technique in more detail. Additionally, the time frames mentioned (1st January 2018 to 31st December 2020 for data, and 1st November 2021 to 1st December 2021 for data collection) should be clearly connected to ensure readers understand the study's temporal framework.

Response to Reviewer comment 4

Quantitative study was conducted using secondary data on pregnancies and deliveries of teenagers in the Asante Akim North District from 1st January 2018 to 31st December 2020. Health facilities and participants were selected using a multi-stage sampling technique. A simple random sampling technique was used to select 4 health facilities for data collection. Data was collected using a structured extraction form and analyzed using descriptive and inferential statistical tools with the help of STATA version 16 at a significant level of 0.05. Chi-square test was performed to determine the relationship between adolescent pregnancies and postpartum haemorrhage.

Reviewers comment 5

Results: The results section provides a clear summary of the findings, highlighting key statistical outcomes. The association between adolescent pregnancies and adverse outcomes is well presented. However, it would be helpful to mention if any comparison was made with early adulthood pregnancies or if the findings were exclusive to adolescents

Response to Reviewer comment 5

The result of the study presented a vivid comparison of early adulthood pregnancies in the discussion.

Reviewers comment 6

Conclusion: The conclusion is succinct but could be strengthened by specifying what kind of reproductive health education is recommended, and by whom (e.g., schools, health facilities, community programs).

Response to Reviewer comment 6

Conclusion: The study found the proportion of adolescent pregnancies and deliveries to be high and therefore requires prompt and effective adolescent reproductive health education. Therefore, it was recommended that through the various District Health Directorates, Ghana Health Service should intensify community and school-based education on effective contraception among sexually active young people to improve their knowledge and understanding of the best use of contraceptives in order to prevent and reduce the rate of adolescent pregnancies and its associated complications.

Reviewer comment 7

Methodology: The abstract suggests a sound methodological approach, but more details in the full text would be necessary to evaluate the robustness of the study. For instance, how was the data validated?
Were there any potential biases in the secondary data used? How was the multi-stage sampling technique implemented, and were there any challenges?

Response to Reviewer comment 7

This study ensured the study's validity by subjecting all the study methods, procedures and tools for data collection to the critical review and approval of the study supervisor and the ethics committee for content validity. The study methods, procedures and data collection tools were also pre-tested in the Asante Akim South District to correct minor errors before the actual study to ensure the reliability of the study. To further enhance the validity and reliability of the study, the

researcher randomly retrieved the mobile numbers of 10% of the study participants and phone calls were made to them. This was to confirm the accuracy of information provided on study participants in their Maternal and Child Health Records Books and what was stated in the records of the health facilities. Also, to avoid biases of the study data incomplete records were replaced with complete ones to ensure internal consistency.

This study adopted a multi-stage cluster sampling technique to recruit participants for the study. The Asante Akim North District has 13 health facilities made up of 1 district hospital, 1 polyclinic, 3 health centers and 8 CHPS compounds. This study excluded all the CHPS compounds within the district because they do not render pregnancy care and delivery services and, therefore, made it impossible to have data on delivery outcomes. A simple random sampling technique was then used to select 4 of the remaining 5 health facilities for data collection. After selecting the facilities, all records of pregnancies and their related outcomes within the defined period of the study; 1st January 2018 – 31st December 2020 constituted the sample frame of the study.

Reviewer comment 8

Statistical Analysis

The use of STATA for data analysis is appropriate. However, the abstract does not specify the statistical tests used to determine significance (e.g., chi-square, t-tests). Including these details would improve transparency

Response to Reviewer comment 8

Inferential statistical was performed using chi-square to test the association between pregnancy outcomes among the young people using the adult group (20 – 24years) as the reference for comparison.

Reviewer comment 9

Results interpretation

The results are statistically significant and relevant to the study’s objectives. It would be beneficial if the abstract mentioned the comparison group (young adults) more explicitly and detailed any differences observed between adolescents and early adults in terms of pregnancy outcomes

Response to Reviewer comment 9

A comparison between the adolescent and early adults have been established in the abstract and the differences observed also stated.

Reviewer comment 10

Language and Clarity

The abstract is generally clear, but there are a few areas where the language could be refined for better readability. For example, the phrase "evidence of maternal of adolescent pregnancies" is unclear and could be revised to "evidence on the maternal outcomes of adolescent pregnancies.

Response to Reviewer comment 10

All issues relating to language and grammatical errors have been corrected and others rephrased for clarity and easy understanding.

Reviewer comment 11

Areas for Improvement: The abstract could benefit from more detailed explanations in certain areas, clearer language, and a stronger conclusion. Additionally, the full article should ensure that all aspects of the methodology, statistical analysis, and discussion are thoroughly explained.

Response to Reviewer comment 11

All reviewers' recommendations regarding the methodology, statistics, and discussion have been fixed.

Thank you for considering our manuscript for publication in PLOS Global Public Health. We look forward to hearing from you soon regarding the outcome of the review process.

Sincerely,

Selina Achiaa Owusu

Corresponding author

Kwame Nkrumah University of Science and Technology

selina.soa@gmail.com
