# Supplementary material for: Comparative analysis of adolescent pregnancy and delivery outcomes versus early adulthood pregnancy in the Asante Akim North District, Ghana
Source: PLOS Glob Public Health. 2025 Feb 24;5(2):e0004290. doi: 10.1371/journal.pgph.0004290 (PMC11849814; doi:10.1371/journal.pgph.0004290)
Supplement: Attachment — Submitted filename: Reviewer comments.pdf [file pgph.0004290.s005.pdf]

Reviewer comments

Title and Keywords

- **Title:** The title is clear and informative, reflecting the content and focus of the study. It specifies the population, location, and comparison, making it relevant for readers interested in adolescent pregnancy and public health in Ghana.
- **Short Title:** The short title is concise and aligns well with the full title.
- **Keywords:** The keywords are appropriate and capture the main aspects of the study. However, they could be expanded to include terms like "maternal health," "reproductive health," and "delivery outcomes."

Abstract

- **Background:** The background is generally clear but could benefit from more detail on the global and regional context of adolescent pregnancy, specifically in LMICs like Ghana. The sentence "Despite the evidence of maternal of adolescent pregnancies globally, little is known, especially in low-income countries" is somewhat awkward and could be rephrased for clarity.
- **Objective:** The objective is clearly stated and aligns with the research question. However, it could be more specific about the types of outcomes being compared (e.g., maternal and neonatal outcomes).
- **Methods:** The methodology is well-outlined, mentioning the study design, data collection period, and tools used for analysis. However, the sentence "Health facilities and participants were selected using a multi-stage sampling technique" could be expanded to explain the sampling technique in more detail. Additionally, the time frames mentioned (1st January 2018 to 31st December 2020 for data, and 1st November 2021 to 1st December 2021 for data collection) should be clearly connected to ensure readers understand the study's temporal framework.
- **Results:** The results section provides a clear summary of the findings, highlighting key statistical outcomes. The association between adolescent pregnancies and adverse outcomes is well-presented. However, it would be helpful to mention if any comparison was made with early adulthood pregnancies or if the findings were exclusive to adolescents.
- **Conclusion:** The conclusion is succinct but could be strengthened by specifying what kind of reproductive health education is recommended, and by whom (e.g., schools, health facilities, community programs).

Methodology

- The abstract suggests a sound methodological approach, but more details in the full text would be necessary to evaluate the robustness of the study. For instance, how was the data validated? Were there any potential biases in the secondary data used? How was the multi-stage sampling technique implemented, and were there any challenges?

Statistical Analysis

- The use of STATA for data analysis is appropriate. However, the abstract does not specify the statistical tests used to determine significance (e.g., chi-square, t-tests). Including these details would improve transparency.

Results Interpretation

- The results are statistically significant and relevant to the study's objectives. It would be beneficial if the abstract mentioned the comparison group (young adults) more explicitly and detailed any differences observed between adolescents and early adults in terms of pregnancy outcomes.

Conclusion and Implications

- The conclusion aligns with the results but could be expanded to discuss potential public health implications and recommendations in more depth.

Language and Clarity

- The abstract is generally clear, but there are a few areas where the language could be refined for better readability. For example, the phrase "evidence of maternal of adolescent pregnancies" is unclear and could be revised to "evidence on the maternal outcomes of adolescent pregnancies."

Suitability for Publication

- **Strengths:** The study addresses an important public health issue and contributes valuable data to the understanding of adolescent pregnancy outcomes in a specific region of Ghana. The methodology and results appear sound and relevant.
- **Areas for Improvement:** The abstract could benefit from more detailed explanations in certain areas, clearer language, and a stronger conclusion. Additionally, the full article should ensure that all aspects of the methodology, statistical analysis, and discussion are thoroughly explained.

Recommendation

- If the full article maintains the quality and addresses the minor issues noted in the abstract, it would be a valuable contribution to the field and could be suitable for publication. However, it would be advisable for the authors to revise the abstract for clarity and completeness before submission.
